# Supplementary material for: Aqueous-Based Synthesis of Photocatalytic Copper Sulfide Using Sulfur Waste as Sulfurizing Agent
Source: Materials (Basel). 2022 Jul 29;15(15):5253. doi: 10.3390/ma15155253 (PMC9369765; doi:10.3390/ma15155253)
Supplement: Supplementary file 1 [file materials-15-05253-s001.zip › materials-1812832-supplementary.pdf]

Article

Aqueous – based synthesis of photocatalytic copper sulfide using sulfur waste as sulfurizing agent

Gabriele Sarapajevaite [1,2,] *, Davide Morselli [2,3] and Kestutis Baltakys [1]

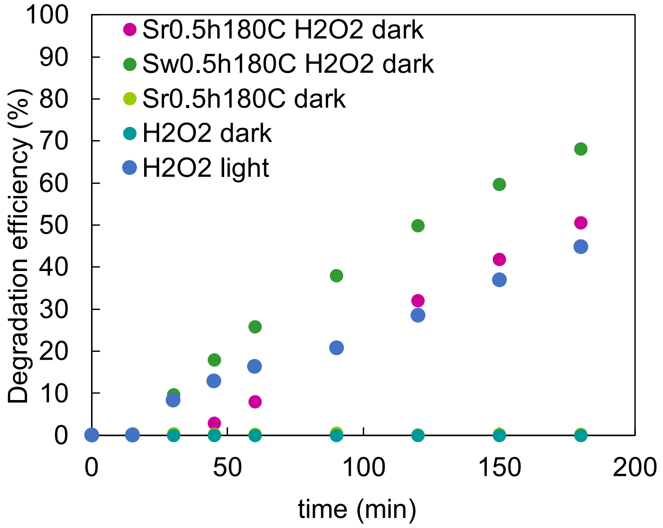

Figure S1. Photocatalytic degradation efficiency of comparison tests with photocatalyst and H_2O_2 in the dark, photocatalyst in the dark, H_2O_2 in the dark and H_2O_2 in the light

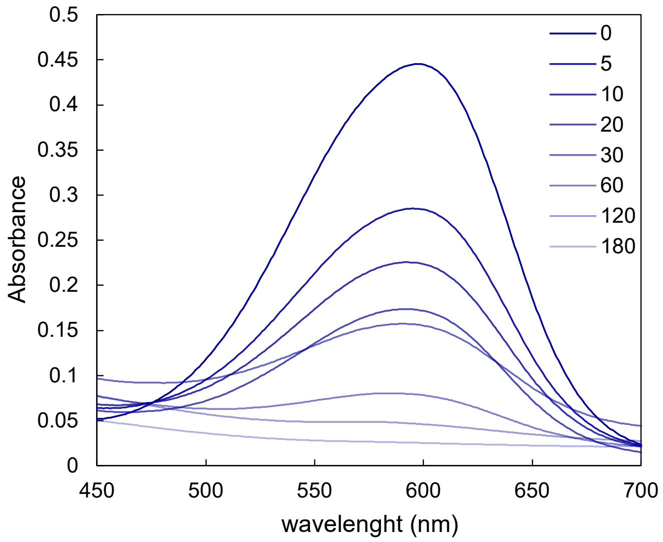

Figure S2. The representative peak of methylene blue spectra during photodegradation for sample Sr0.5h180C
